# Arthroscopic treatment of unstable scaphoid fracture and nonunion with two headless compression screws and distal radius bone graft

**DOI:** 10.1186/s13018-023-03529-7

**Published:** 2023-01-18

**Authors:** Cong Cheng, Zongyuan Jiang, Haoran Sun, Jiaping Hu, Yanggang Ouyang

**Affiliations:** Department of Hand Surgery, Affiliated Longhua People’s Hospital, Southern Medical University, Shenzhen, 518000 China

**Keywords:** Scaphoid fracture, Nonunion, Headless compression screw, Bone graft, Arthroscopy

## Abstract

**Background:**

The treatment of unstable scaphoid fracture and nonunion remains a challenging problem for hand surgeons. Minimally invasive treatment has become the preferred method of treatment.

**Purpose:**

This study introduces the arthroscopic technique with two headless compression screws (HCS) fixation and distal radius bone grafting for the treatment of unstable scaphoid fracture and nonunion, aiming to evaluate its clinical and radiological outcomes.

**Methods:**

It was a retrospective study. From January 2019 to February 2021, a total of 23 patients were included in the current study. Among them, 13 patients with unstable scaphoid fracture underwent arthroscopic treatment with two HCS; 10 patients with scaphoid nonunion underwent arthroscopic treatment with two HCS and a distal radius bone graft. The range of motion of the wrist, visual analog scale (VAS), grip strength, the Modified Mayo Wrist Score (MMWS), the Patient-Rated Wrist Evaluation (PRWE) score, and the Disability of the Arm, Shoulder and Hand (DASH) score were collected at preoperatively and the final follow-up. A computed tomography scan of the wrist was performed on each patient to analyze for union and postoperative osteoarthritis during the follow-up period.

**Results:**

Significant improvement was only observed in wrist extension. Clinical outcomes including grip strength, VAS pain score, MMWS, PRWE score, and DASH score were significantly improved at the final follow-up. In the subgroup analysis, both patients stabilized with either two HCS or a distal radius bone graft and two HCS have improved clinical outcomes after surgery, respectively. All patients achieved union. No screw fixation failure occurred, and no other postoperative complication was observed in any of the patients.

**Conclusions:**

The arthroscopic technique with two-HCS fixation and distal radius bone grafting is a reliable and effective technique for the treatment of unstable scaphoid fracture and nonunion, providing satisfactory union rates and clinical outcomes.

## Background

The scaphoid is the most commonly fractured carpal bone. Scaphoid fracture accounts for 60–80% of all carpal fractures, and 2–3% of all fractures [1], which mainly occur in young and active individuals as a result of a fall on an ulnarly deviated, outstretched, and pronated hand [2]. Untreated or improperly treated scaphoid fracture is prone to cause nonunion, avascular necrosis, flexion deformity, progressive deterioration of articular cartilage, and scaphoid nonunion advanced collapse (SNAC) [3]. The scaphoid has a unique anatomy. Approximately 80% of its surface is covered by cartilage. Only a small surface area of the scaphoid is available for the inflow of the blood supply which originates from the dorsal branches of the radial artery traveling in a predominantly retrograde manner [4]. The management of scaphoid fracture depends largely on the fracture location and displacement. According to the Herbert classification, scaphoid tubercle fractures and incomplete waist fractures (Type A) are stable, which can potentially be treated conservatively. Oblique fractures of the distal third, displaced/mobile fractures of the waist, proximal pole fractures, fracture dislocations, and comminuted fractures of the scaphoid (Type B) are considered unstable and should be treated with surgical fixation [3, 5].

Surgical treatment of scaphoid fracture with a headless bone screw was introduced by Herbert and Fisher in 1984 [6]. The screw was subsequently modified by Whipple and other orthopedic surgeons into a headless compression screw (HCS) in the 1990s, making percutaneous fixation possible [7, 8]. Trumble et al. [9] proposed that a secondary derotational screw was the decisive factor to improve rotational stability and union rate. Slade et al. [10] supported the use of additional implants within the scaphoid for augmentation of the central screw construct. A retrospective case series of 25 patients found that the two-screw fixation for scaphoid fractures achieved a 100% union rate and Mayo Wrist Score of greater than 90 at the final follow-up, and time to return to activity decreased with confidence in a stronger biomechanical construct [11]. Thus, two-HCS fixation appeared to be technically effective and safe, and also highly demanding. Minimally invasive treatment for scaphoid fracture and nonunion has garnered considerable interest among hand surgeons because of its advantages of faster recovery and less complications. Several studies have been published reporting promising results under arthroscopic treatment with one HCS and an optional bone graft [10, 12–15].

The purpose of this present study was to assess the clinical and radiological outcomes of a consecutive single-surgeon series of patients with unstable scaphoid fracture and nonunion who underwent two-HCS fixation and distal radius bone grafting under arthroscopy. We hypothesized that this technique could result in satisfactory outcomes by allowing for early active range of motion (ROM) and enhanced recovery after surgery.

## Patients and methods

**Fig. 1:**
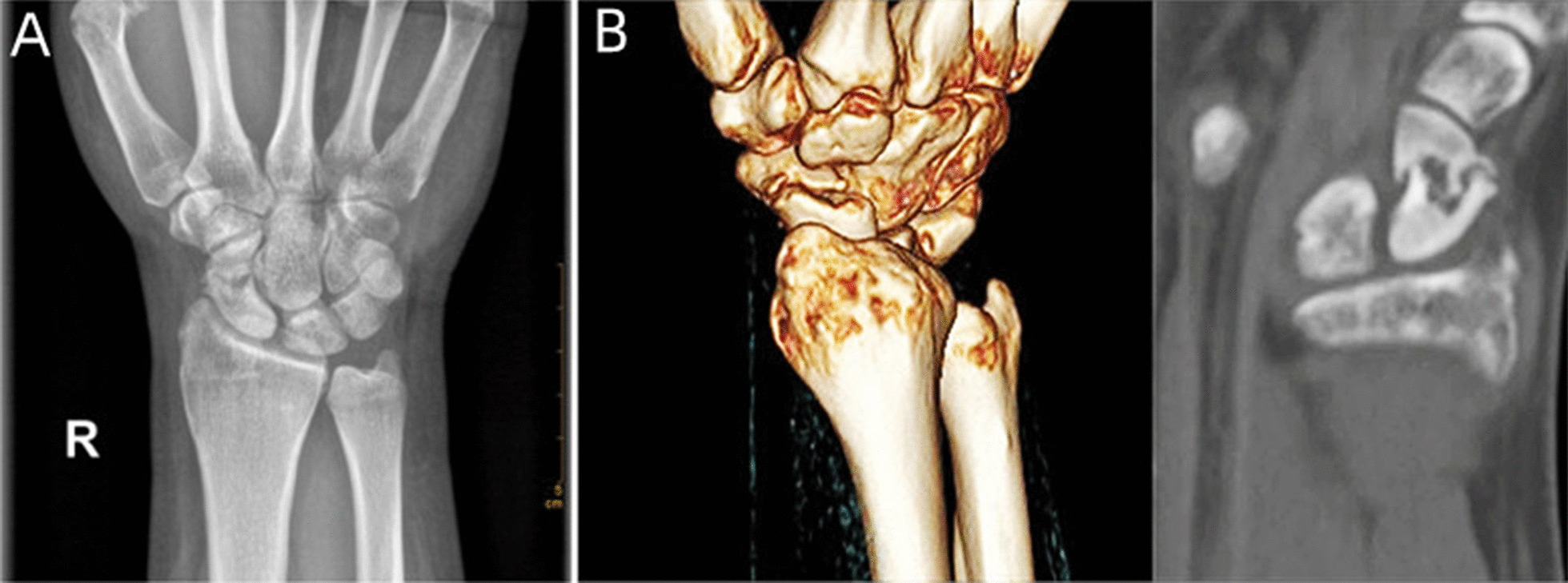
**A** The preoperative plain radiograph of scaphoid nonunion in a 26-year-old man; **B** A reformatted CT scan shows the deformity of the sclerotic scaphoid nonunion

The Institutional Committee on Ethics in our institution approved this retrospective follow-up study. Informed consent was obtained from all patients preoperatively. Between January 2019 and February 2021, the clinical data of patients with scaphoid fracture or nonunion were collected. Inclusion criteria were: (1) age above 18 years, (2) unstable scaphoid fracture [5], (3) scaphoid delayed nonunion and nonunion (defined as no evidence of healing within 3 months and more than 6 months after the initial injury, respectively) (Fig. 1), (4) more than 12 months of follow-up, and (5) treatment by two-HCS fixation with or without an autologous distal radius bone graft under arthroscopy. Exclusion criteria were: (1) bone absorption and loss of height with humpback deformity of the scaphoid, (2) AVN of the proximal pole, (3) Mack–Lichtman type IV and V scaphoid nonunions [16], (4) local or systemic arthritis involving the affected wrist, (5) previous injuries or surgical treatment of the affected wrist, and (6) wrist deformity. All included patients were operated in our institution, and all surgical procedures were performed by one senior author that specialized in wrist arthroscopy.

## Surgical technique

The patient was placed in a supine position under regional anesthesia with the surgical arm on a radiolucent hand table and a nonsterile tourniquet. The arm was prepared and draped to above the elbow in a standard sterile fashion, and the tourniquet was inflated to 250 mmHg.

The arm was then placed in a vertical wrist traction tower. Ten to fifteen lbs traction was applied through plastic finger traps to the index, middle, and ring fingers. The standard 3–4 and 4–5 portals were established. The radiocarpal and ulnocarpal joints were inspected routinely to detect intraarticular pathologies with a 2.3-mm arthroscope. If there were synovitis, cartilage degeneration, triangular fibrocartilage complex (TFCC) and intercarpal ligament injuries, synovectomy, debridement of the injured cartilage, radial styloidectomy, and TFCC repair were performed.

**Fig. 2:**
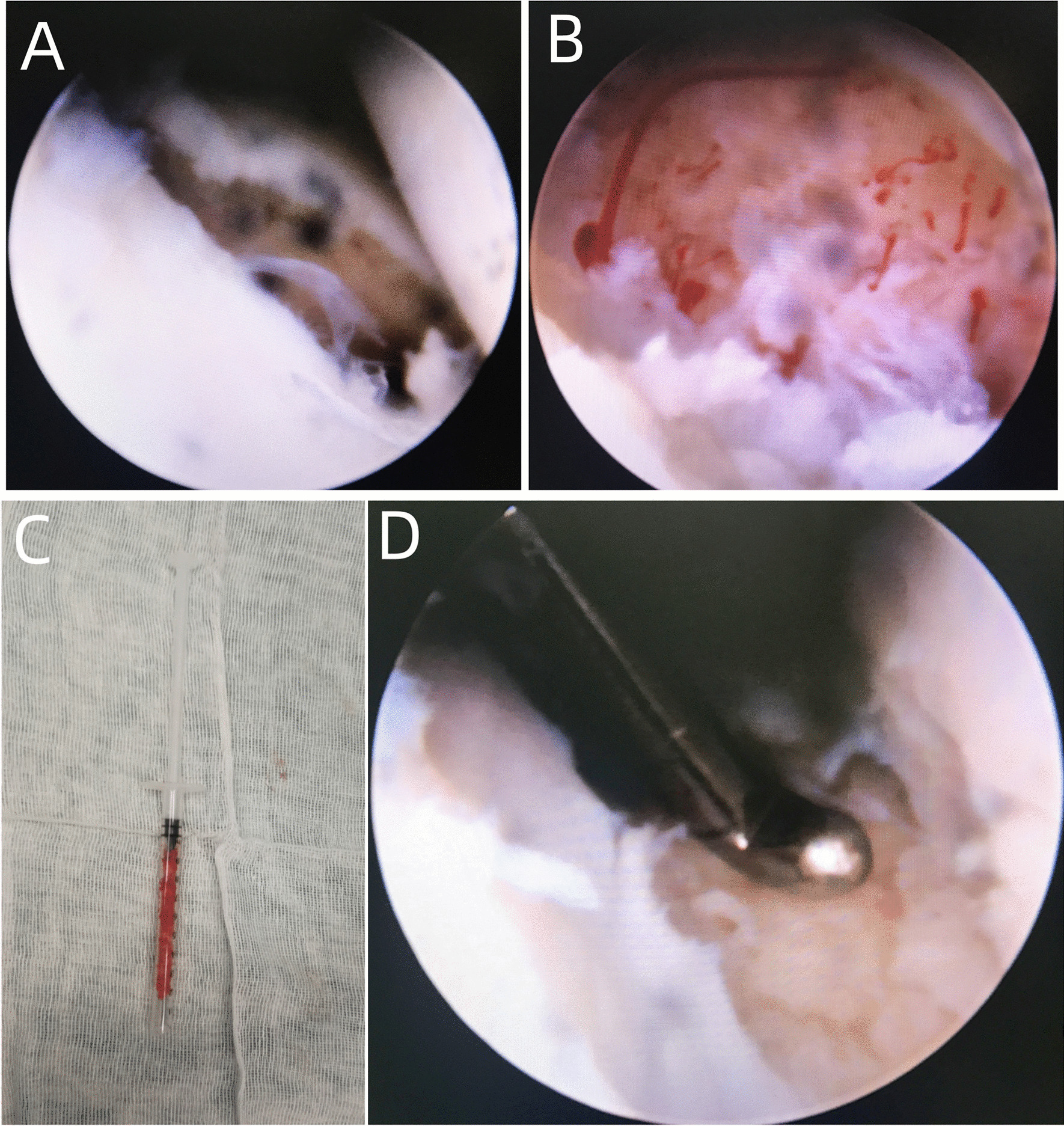
**A** Arthroscopic view of cleavage line in scaphoid articular surface at the nonunion site. **B** Punctate bleeding after adequate debridement. **C** Bone graft harvested from the distal radius is cut into small pieces and placed into a 1-mL syringe. **D** Bone graft is impacted into the defect site

The midcarpal ulnar (MCU) portal was then established to visualize the scaphoid fracture and nonunion site (Fig. 2A), while the midcarpal radial (MCR) portal and scaphotrapeziotrapezoid (STT) portal were established for working. At the nonunion site, the fibrotic tissue, bony callus and sclerotic bone were debrided using a motorized 2.9-mm shaver, fine-angled curette, and 2.9-mm burr through the MCR portal, until fresh cancellous bone was evident in both the proximal and distal fragments. After that, the tourniquet was deflated to assess punctate bleeding from the fragments (Fig. 2B).

A 2-cm longitudinal incision was made in the second dorsal compartment proximal to the distal radius. Blunt dissection was carried down, and the bony surface was fully exposed. A 1-cm osteotome was used to create and open a cortical window, followed by an angled curette for harvesting cancellous bone graft. The bone graft was subsequently cut into small pieces, placed into a 1-mL syringe, and compressed with the plunger (Fig. 2C). The cortical window was then reapproximated to prevent subcutaneous hematoma.

To correct scaphoid alignment, two 1.2-mm Kirschner wires were inserted perpendicularly into both proximal and distal fragments as “joysticks.” With wrist extension and ulnar deviation, the fracture fragments were manipulated by the joysticks, and a probe was simultaneously used to facilitate the reduction through the MCR portal, until the fragments were well aligned, and no rotational deformation and articular stepping were detected under both arthroscopy and fluoroscopy.

**Fig. 3:**
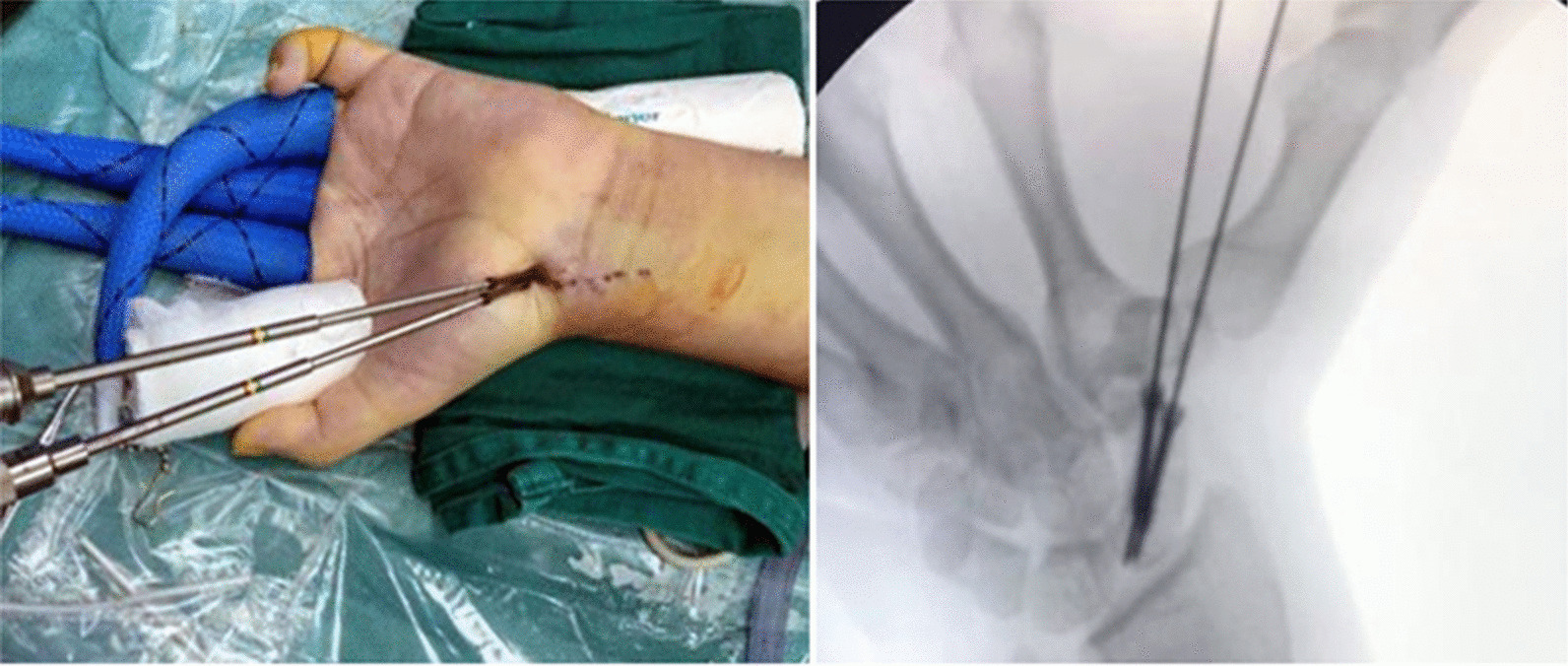
**A** Two headless compression screws are inserted along the axial of the K-wires and advanced alternately. **B** Two screw heads are confirmed to be positioned beneath the articular surface of scaphoid under fluoroscopic examination

Over the scaphoid tuberosity, a 1.0-mm K-wire was percutaneously drilled into the distal pole of scaphoid, which was placed in a retrograde fashion along the central axis of the scaphoid under fluoroscopic guidance until the subchondral bone of the proximal pole was reached. After determining the correct position, the 1-mL syringe was placed through the MCR or STT portal into the defect site, while the arthroscope was placed through the MCU portal to visualize grafting. The bone graft was then inserted and manually impacted into the defect site (Fig. 2D). After completeness and adequacy of the graft filling, another equal-length K-wire was drilled. Of note, the two K-wires should be preferably placed parallelly. With proper length measured by a gauge, two screws (diameter, 2.4 mm) were subsequently inserted over the K-wires, and advanced alternately in order to avoid the risk of an asymmetrical reduction and compression (Fig. 3A). The two K-wires were then removed once two screw heads were confirmed to be positioned beneath the articular surface of scaphoid under fluoroscopic examination (Fig. 3B). Patients with unstable scaphoid fracture were treated with two-HCS fixation arthroscopically as mentioned above, without bone grafting.

## Postoperative management

All patients underwent similar rehabilitation programs. They were immobilized with a below elbow thumb spica plaster splint for 2 weeks. At the first postoperative appointment, patients with a removable spica plaster commenced early active ROM of the wrist and hand. Initiation of full ROM and gentle grip strengthening exercises started at postoperative 3 weeks. Lifting and holding exercises were allowed at postoperative 1 month. Patients were progressed to daily activities and non-contact sports at postoperative 2–3 months once radiographic union is confirmed. Full axial loading was encouraged at 3 months after surgery. All patients followed up for at least 12 months.

## Follow-up evaluation

The assessment included preoperative and postoperative radiographic and clinical examinations by two hand surgeons. The results of CT scans of the injured wrists were evaluated according to the following criteria: the union was defined as more than 50% of trabecular bridging on three or more slices of the scan on the radiographs. CT scans were reconstructed along the longitudinal axis of the scaphoid to assess the fracture union at the follow-up, and union time was recorded when consolidation at the fracture site was confirmed finally. The final follow-up CT scans were examined to classify postoperative osteoarthritis (POA) as stage 0 (none), stage 1 (mild breaking of the radius without the involvement of the radioscaphoid joint), stage 2 (narrowing of the radioscaphoid joint space), and stage 3 (loss of the radioscaphoid joint space) [17]. Fracture type based on the modified Herbert classification was evaluated by CT scans [5]. The location of fracture was also recorded, which was classified as proximal, waist and distal.

The following data were collected for analysis: age, gender, dominant hand, time to operation, surgical technique, follow-up time, visual analog scale [VAS; ranging from O (no pain) to 10 (worst possible pain)], grip strength in kilograms (Jamar, Sammons Preston Rolyan, Mississauga, Ontario, Canada). Patient-reported outcome measures were obtained including the Modified Mayo Wrist Score (MMWS) (0–100 points), the Patient-Rated Wrist Evaluation (PRWE) score (0–100 points), and the Disability of the Arm, Shoulder and Hand (DASH) score (0–100 points). The ROM was evaluated with a standard clinical goniometer. All variables were re-evaluated at the final follow-up by an independent evaluator. Postoperative complications were also recorded.

Statistical analyses were performed with SPSS software version 22.0 (SPSS Inc., Chicago, IL, USA). The data of outcome measures were given as mean, standard deviation, and 95% confidence interval. The paired t test was used to compare the preoperative and postoperative outcome data. A p value less than 0.05 was considered statistically significant.

## Results

**Fig. 4:**
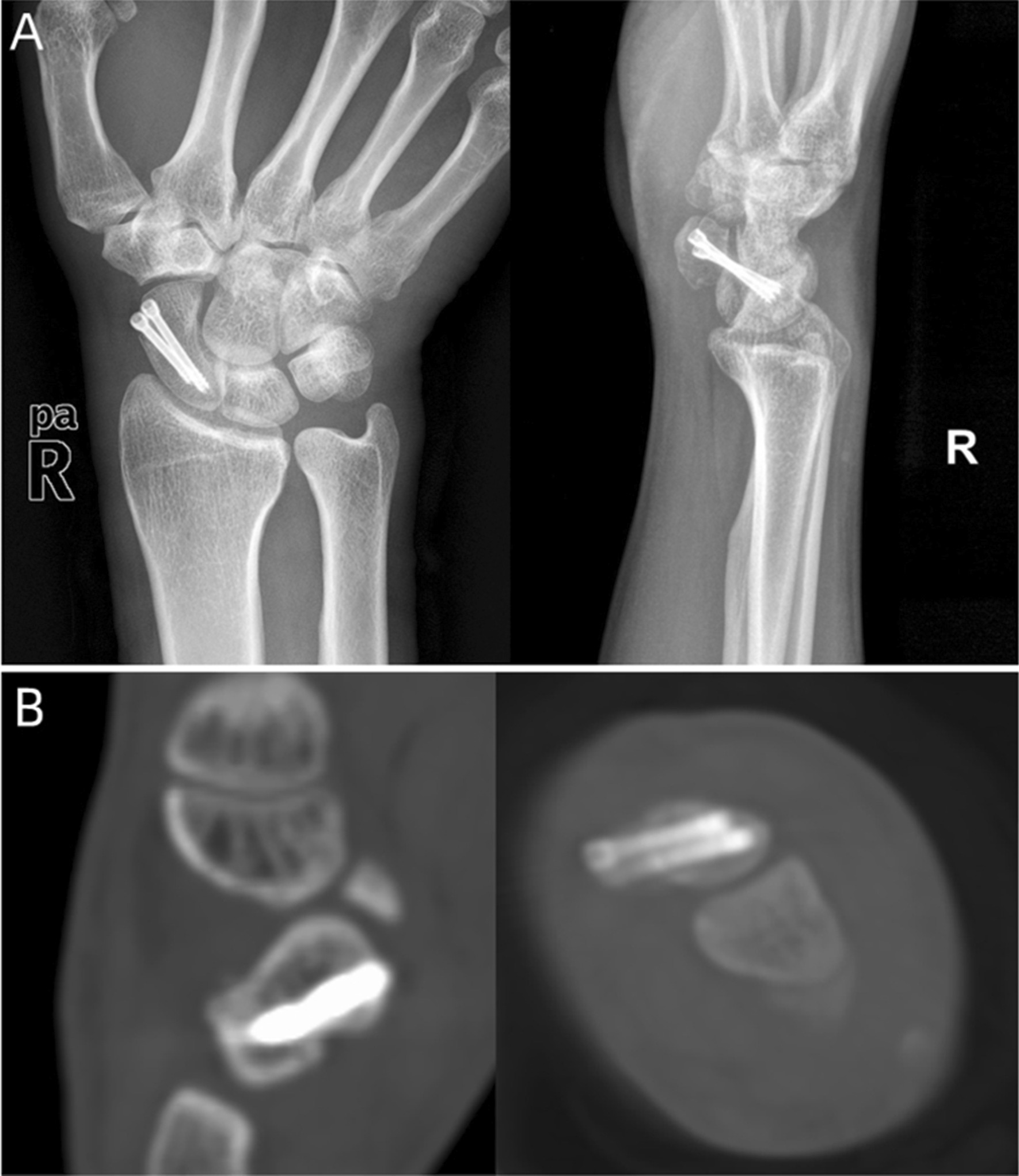
Union was achieved at 12 weeks after surgery, which was assessed by plain radiograph (**A**) and CT scan (**B**)

A total of 32 patients with scaphoid fracture or nonunion were treated surgically at our institution during the study period. Of these, 7 patients were not eligible according to the inclusion criteria, and 2 patients refused to participate in the follow-up investigation. Therefore, 23 patients (16 males and 7 females) were included in the study with a mean age of 30.9 years (range, 19–49 years). Among them, 13 patients were diagnosed with unstable scaphoid fracture and underwent arthroscopic treatment with two HCS (age range, 21–49 years); 10 patients were diagnosed with scaphoid nonunion and underwent arthroscopic treatment with two HCS and a distal radius bone graft (age range, 19–43 years). The average time between initial injury and surgery of the HCS group and the bone graft with HCS group was 4.3 days (range, 1–14 days) and 15.1 months (range, 4–84 months), respectively. The dominant wrist was injured in 18 cases. The mean follow-up time was 14.8 months (range, 12–18 months). According to Herbert classification, 7 cases were B2, 5 were B3, 3 were C, 2 were D1, and 6 were D2. The proximal fracture was found in 8 cases, and waist fracture in 15 cases. With respect to POA, 17 cases were graded as stage 0, and 6 as stage 1. At the final follow-up, all patients had achieved bone healing at an average of 11.6 weeks (range, 8–20 weeks) (Fig. 4), among which the HCS group was 10 weeks (range, 8–12 weeks), and the bone graft with HCS group was 13.2 weeks (range, 10–20 weeks). No patient had nonunion, scaphoid deformity or screw fixation failure that required revision surgery. Detailed principal patient characteristics are presented in Table 1.

**Table 1:** Principal patient characteristics

Group	Pt. no.	Age (years)	Sex	Hand dominance	Location of fracture/union	Herbert classification	Time to surgery (d and m)	POA stage	Bony union (weeks)	Follow-up (months)
HCS (*n* = 13)	1	34	M	Dominant	Waist	B2	1 d	0	8	18
	2	28	F	Dominant	Proximal	B3	1 d	0	10	18
	3	35	M	Dominant	Proximal	B3	3 d	0	12	14
	4	26	F	Nondominant	Waist	B2	1 d	0	8	16
	5	49	F	Dominant	Proximal	B3	2 d	0	10	12
	6	26	M	Nondominant	Waist	B2	14 d	0	12	14
	7	48	F	Dominant	Waist	B2	1 d	0	8	16
	8	25	M	Dominant	Waist	B2	5 d	0	10	12
	9	21	F	Nondominant	Proximal	B3	2 d	0	8	12
	10	33	M	Dominant	Proximal	B3	1 d	0	12	16
	11	37	M	Nondominant	Waist	B2	14 d	0	10	12
	12	30	M	Nondominant	Waist	B2	4 d	0	10	16
	13	28	M	Dominant	Waist	B2	7 d	0	12	12
	Mean	32.3					4.3 d		10	14.5
Bone graft with HCS (n = 10)	14	23	M	Dominant	Proximal	C	4 m	0	12	16
	15	26	M	Dominant	Waist	D1	6 m	0	12	18
	16	43	M	Dominant	Proximal	C	5 m	1	10	16
	17	23	M	Dominant	Waist	D2	84 m	1	20	14
	18	36	M	Dominant	Waist	D2	12 m	1	12	16
	19	35	M	Dominant	Waist	D2	8 m	1	14	12
	20	21	M	Dominant	Waist	D1	6 m	0	12	12
	21	40	M	Dominant	Waist	C	4 m	0	14	14
	22	19	F	Dominant	Waist	D2	12 m	1	12	18
	23	26	F	Dominant	Proximal	D2	10 m	1	14	14
	Mean	29.2					15.1 m		13.2	15
Total (n = 23)	Mean	30.9					6.9 m		11.6	14.8

*M* male, *F* female, *HCS* headless screw, *POA* postoperative osteoarthritis

**Fig. 5:**
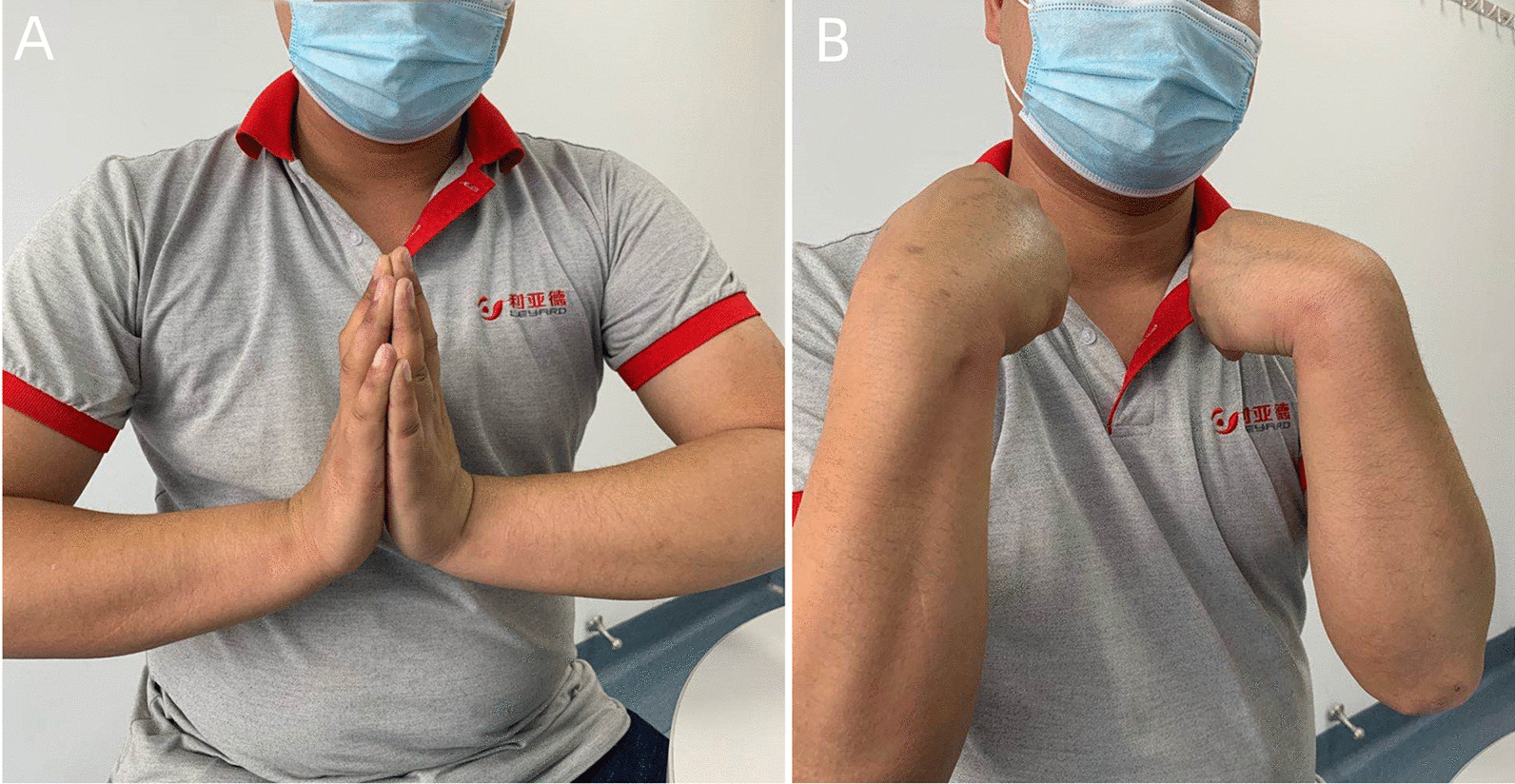
**A** Range of motion of the wrist is excellent at 12 months after surgery. **B** The scars are inconspicuous

Significant improvement was only observed in wrist extension. Clinical outcomes including grip strength, VAS pain score, MMWS, PRWE score, and DASH score were significantly improved at the final follow-up. In the subgroup analysis, both patients stabilized with either two HCS or a distal radius bone graft and two HCS have improved clinical outcomes after surgery, respectively. No patient reported any postoperative complications at the final follow-up, including chronic pain, infection, and donor site-related problems. All patients returned to their regular activities (Fig. 5). Preoperative and postoperative outcome parameters are given in Table 2.

**Table 2:** Preoperative and postoperative outcome parameters

Group	HCS (*n* = 13)	Bone graft with HCS (*n* = 10)	Total (*n* = 23)
*Flexion*			
Preoperative	54.33 ± 10.67	52.86 ± 4.42	53.54 ± 7.98
Postoperative	58.50 ± 7.09	57.29 ± 12.60	57.85 ± 10.44
Mean difference (95% CI)	4.17 (− 6.08 to 14.42)	4.43 (− 5.46 to 14.43)	4.30 (− 2.84 to 11.44)
*P* Value	0.484	0.432	0.267
*Extension*			
Preoperative	48 ± 10.16	50.29 ± 11.89	49.23 ± 11.18
Postoperative	64.01 ± 9.15	63.57 ± 7.69	63.77 ± 8.40
Mean difference (95% CI)	16.01 (5.07 to 26.95)	13.28 (2.79 to 23.77)	14.54 (6.94 to 22.14)
*P* Value	0.03*	0.04*	0.001*
*Ulnar deviation*			
Preoperative	29.71 ± 12.68	24.17 ± 12.68	27.15 ± 12.28
Postoperative	33.14 ± 7.53	30.50 ± 7.53	31.92 ± 10.39
Mean difference (95% CI)	3.43 (− 8.37 to 15.23)	6.33 (− 4.59 to 17.25)	4.77 (− 3.97 to 13.51)
*P* Value	0.580	0.422	0.315
*Radial deviation*			
Preoperative	16.17 ± 3.39	15.86 ± 3.44	16 ± 3.42
Postoperative	19 ± 3.21	18.86 ± 4.61	18.92 ± 4.03
Mean difference (95% CI)	2.83 (− 0.91 to 6.57)	3 (− 1.26 to 7.26)	2.92 (0.05 to 5.79)
*P* Value	0.205	0.226	0.067
*VAS pain score*			
Preoperative	5.6 ± 1.2	5.57 ± 1.4	5.58 ± 1.32
Postoperative	1.4 ± 1.02	1.71 ± 0.88	1.6 ± 0.95
Mean difference (95% CI)	− 4.2 (− 2.82 to − 5.58)	− 3.86 (− 2.64 to − 5.08)	− 3.98 (− 3.06 to − 4.90)
*P* Value	0.001*	< 0.001*	< 0.001*
*DASH score*			
Preoperative	28 ± 3.03	27.29 ± 3.01	27.6 ± 3.04
Postoperative	3.2 ± 0.98	5.57 ± 1.92	4.48 ± 1.98
Mean difference (95% CI)	− 24.80 (–27.59 to − 22.01)	− 21.72 (− 24.36 to − 19.08)	− 23.12 (− 25.17 to − 21.07)
*P* Value	< 0.001*	< 0.001*	< 0.001*
*PRWE score*			
Preoperative	53.6 ± 8.73	53.71 ± 10.26	53.67 ± 9.66
Postoperative	9 ± 1.41	9.43 ± 2.19	9.25 ± 1.92
Mean difference (95% CI)	− 44.60 (− 52.32 to − 36.88)	− 44.28 (− 52.05 to − 36.51)	− 44.42 (− 49.99 to − 38.83)
*P *Value	< 0.001*	< 0.001*	< 0.001*
*MMWS*			
Preoperative	56 ± 9.25	61.71 ± 6.20	59.33 ± 8.13
Postoperative	88.8 ± 6.55	83.57 ± 11.94	85.75 ± 10.34
Mean difference (95% CI)	30.60 (26.01 to 35.19)	41.58 (38.83 to 44.33)	37.02 (33.56 to 40.48)
*P* Value	< 0.001*	0.001*	< 0.001*
*Grip strength, kg*			
Preoperative	30.1 ± 2.97	27 ± 3.34	28.25 ± 3.51
Postoperative	41.8 ± 4.83	42.86 ± 4.91	42.41 ± 4.83
Mean difference (95% CI)	11.70 (16.67 to 6.73)	15.86 (11.46 to)	14.16 (10.78 to 17.54)
*P* Value	0.003*	< 0.001*	< 0.001*

Data are presented as mean ± standard deviation

*CI* confidence interval, *DASH* Disabilities of the Arm Shoulder and Hand, *VAS* visual analog scale, *PRWE* Patient-Rated Wrist Evaluation, *MMWS* Modified Mayo Wrist Score

*P < 0.05

## Discussion

The management of scaphoid fracture and nonunion is still challenging for hand surgeons. If not treated properly, it will lead to considerable consequences in patients' wrist function. The goal of treatment is to achieve union, correct the deformity, relieve pain, improve function, and prevent the progression of osteoarthritis [2, 3]. In our study, the arthroscopic treatment of displaced scaphoid fracture and nonunion using two-HCS fixation and a distal radius bone graft has provided satisfactory results, which were composed of a union rate of 100%, and significantly improved clinical outcomes.

The difference in union rates for conservative treatment of scaphoid fractures may be due to the combination of heterogeneous patient groups with different fracture types and locations. Gellman et al. [18] reported a union rate of greater than 95% of nondisplaced scaphoid fractures treated with casting immobilization. Bond et al.[19] showed a 100% union rate of nondisplaced scaphoid fractures with casting immobilization, but longer radiographic healing time compared with surgical fixation. Several other comparative studies in patients with acute scaphoid waist fractures demonstrated better ROM of the wrist, grip strength, faster healing time, and earlier return to work with surgical fixation [20, 21]. A meta-analysis involving 378 patients with nondisplaced or minimally displaced scaphoid fractures reported that surgical fixation could result in decreased rates of delayed union, and decreased time away from work [22]. However, another meta-analysis documented that patients treated surgically had an odds ratio of 6.96 for complications compared with patients treated nonsurgically [23]. The long-term risks and benefits of surgical intervention should be carefully weighed and considered when managing the scaphoid waist fractures.

Biomechanical studies demonstrated that multidirectional movement of the scaphoid generated bending, rotational and translational forces at the fracture site, thereby exerting a negative impact on bone healing and giving rise to fibrous tissue formation [24, 25]. Rigid fixation was definitely of great importance to resist these forces and facilitate primary bone healing. Reliable union rates and good functional outcomes were reported in patients treated with volar plate fixation [26, 27]. However, it was suggested that this technique has significant complications such as plate breakage, screw backout, plate impingement, inadequate compression across the fracture site as well as higher costs [28]. Recently, two-HCS fixation has been advocated after extensively biomechanical investigating on fixation methods. A biomechanical study of Acar et al. using three-dimensional finite element analysis in three different wrist positions declared that two-HCS fixation imparted less displacement of the fracture gap and lower rotation of the fracture fragments than one-HCS fixation [29]. Mandaleson et al. [30] compared three fixation options in a scaphoid nonunion model and reported that both double HCS and plate fixation had significantly greater stability, stiffness, and energy absorption than one-HCS fixation. No significant differences could be observed between two-HCS fixation and scaphoid plate fixation in any of the biomechanical parameters. The theoretical advantage of this technique is to achieve multiplanar stability by maximizing interfragmentary compression across the fracture site while minimizing shear and rotational forces that may hinder primary bone healing [31].

Garcia et al. [32] presented 19 patients with scaphoid nonunion using two-HCS fixation, showing superior rotational stability and a union rate of 100% without screw penetration and postoperative complications. Quadlbauer et al. [33] included a total of 47 patients with unstable scaphoid B2 type fracture and demonstrated that patients treated with two HCS had a significantly higher union rate than those with one HCS. EK et al. [34] showed good clinical outcomes and high union rates (90.5%) with two-HCS fixation and autologous distal radius cancellous bone grafting for delayed scaphoid unions and nonunions in a series of 21 patients. A retrospective study of 42 patients with scaphoid nonunions fixed with one versus two HCS found that two-HCS fixation had a higher union rate [35]. With regard to screw length, Dodds et al. [36] suggested that the long screw was superior to the short one when checked for fracture fragment stability. We used two HCS measuring 18–26 mm for securing the fractures. We believe that the rigid fixation allows for early rehabilitation. Thus, all patients were advised to remove spica plaster and encouraged to initiate active ROM in the early stage after surgery.

Minimally invasive techniques are primarily indicated for minimally or nondisplaced scaphoid waist fractures and proximal pole fractures. A displacement greater than 1.0 mm is an indication of open reduction to obtain the anatomic alignment. However, these techniques have been successfully applied to the displaced scaphoid fractures, which usually require significant manipulation to correct the alignment. When this closed reduction cannot be achieved, open reduction is required. But the probability of such cases is relatively low [37]. Moreover, minimally invasive techniques are not applicable to all types of scaphoid nonunions. They are indicated for early scaphoid nonunions without substantial bone absorption and loss of height with humpback deformity, and without AVN of the proximal pole. The chronic scaphoid nonunions with established structural deformities should be treated with more complicated procedures, usually involving open reduction, bone grafting and internal fixation [38]. Although these techniques are successful, a thorough preoperative discussion is essential. Both the surgeons and patients must be aware of the risks of inadequate reduction and further redisplacement [39].

Slade et al. [10] first described the use of one percutaneous screw fixation under arthroscopy for the treatment of scaphoid nonunion. They reported good results with a union rate of 100% and a comparable ROM and grip strength of 87% compared to the contralateral hand at 1-year follow-up. Waitayawinyu et al. [13] studied a consecutive cohort of 22 patients treated with one HCS and an olecranon bone graft under arthroscopy, and all patients had satisfactory outcomes and bone healing. Wong et al. [14] found an overall union rate of 90.7% after arthroscopic treatment with one HCS and an iliac crest bone graft. The arthroscopic technique with two-HCS fixation and optional bone grafting has not been discussed to date. We recognize that the arthroscopic technique has several advantages. First, arthroscopy helps to visualize the fracture site and assess the fracture features. Adequate freshening of the fracture site could be easily performed, and punctate bleeding is also clearly visible. Second, concomitant injuries could be diagnosed under arthroscopic examination and treated at the same time. Third, external joystick reduction in the proximal and distal fracture fragments could be facilitated by the use of an arthroscopic probe. Moreover, it helps to visualize directly whether there is screw penetration above the articular surface of the scaphoid. It also contributes to minimizing surgical trauma to ligamentous structure and blood supply, less dissection of the soft tissue and the capsule, allowing for early restoration of wrist function. Additionally, it requires smaller skin incisions.

The gold standard for treating scaphoid nonunion is autogenous bone grafting. A randomized control trial reported similar results regarding union rate, time to union, and functional outcomes in 80 patients who received either vascular or nonvascular bone grafts, but three patients involved graft failure in the vascular bone graft group [40]. The vascular bone grafting demanding microvascular procedures was complex, which required longer operation time, extensive dissection, limited pedicle length, and difficulty in obtaining and placing the graft [41, 42]. Nonvascularized bone grafts from the iliac crest and distal radius are most commonly used in scaphoid nonunion and proved to provide osteoconductive and other progenitor cells for accelerating bone healing [43]. Garg et al. [44] conducted a prospective randomized trial of 100 patients to compare union rate and functional outcome between the two graft types. No significant difference was observed with a union rate of 87% for both. Tambe et al. [45] investigated 68 symptomatic scaphoid nonunion patients; the two graft types provided an equivalent union rate and radiologic outcomes, but a donor-site pain was found in 21% of patients undergoing iliac crest bone grafting. In a large systematic review by Pinder et al. [46] involving 1602 patients, they revealed that iliac crest bone graft had a comparable union rate with distal radius bone graft (87% and 89%, respectively), but a higher incidence of complications. We advocate the use of distal radius bone grafts as they are minimally invasive and require only regional anesthesia while iliac crest bone grafting usually requires general anesthesia. Furthermore, harvesting bone graft from the distal radius may increase the vascularity of the area and generate a form of regional bone repair response, as a result, improving the vascular flow in the wrist. This theoretical framework was proposed by Rellan et al. [47], where core decompression of the distal radius using an antegrade screw was proven to be successful in treating scaphoid nonunion. Our technique harvested adequate graft material from the distal radius to fill in the fracture site. Union was achieved in all 23 cases at an average of 11.6 weeks.

Besides the nonunion location and fixation stability, time to surgery was perceived as another major risk factor that resulted in the failure of treatment. Mahmoud et al. [48] reported that the average healing time was 9 weeks if time to surgery was less than 1 year, whereas an average healing time of 12 weeks was needed to achieve union if time to surgery was greater than 1 year. A multivariable regression analysis found a significant correlation between union rate and time to surgery in 160 scaphoid nonunion cases (a union rate of 92% if the nonunion duration was less than 5 years versus 80% if the nonunion duration was greater than 5 years) [49]. It seemed plausible that progressively greater failure rates were associated with longer delays. Our study included patients with a wide range of time to surgery (from 1 day to 84 months). However, all patients achieved bony union with different healing time, which may prove that our technique is a valid treatment option for unstable scaphoid fracture and nonunion.

Several limitations of our study should be considered prior to interpretation. There was no control group including other techniques such as one HCS for a comparison with the experimental group. It is a retrospective follow-up investigation with a nonrandomized patient population, which may lead to selection bias. The small number of patients and the short-term clinical and radiographic follow-ups also limit the strength of our findings. We recognize that further high-quality studies with larger sample sizes and long-term follow-ups are warranted to determine the effectiveness and safety of this technique.

In conclusion, the arthroscopic technique with two-HCS fixation and distal radius bone grafting is a reliable and effective technique for the treatment of unstable scaphoid fracture and nonunion, providing satisfactory union rates and clinical outcomes.

## Abbreviations

HCS: Headless compression screw
ROM: Range of motion
VAS: Visual analog scale
MMWS: Modified Mayo Wrist Score
PRWE: Patient-Rated Wrist Evaluation
DASH: Disability of the Arm, Shoulder and Hand
CT: Computed tomography
SNAC: Scaphoid nonunion advanced collapse
TFCC: Triangular fibrocartilage complex
MCU: Midcarpal ulnar
MCR: Midcarpal radial
STT: Scaphotrapeziotrapezoid
POA: Postoperative osteoarthritis
